# Mesenchymal stem cell therapy for severe COVID-19

**DOI:** 10.1038/s41392-021-00754-6

**Published:** 2021-09-08

**Authors:** Lei Shi, Lifeng Wang, Ruonan Xu, Chao Zhang, Yunbo Xie, Kai Liu, Tiantian Li, Wei Hu, Cheng Zhen, Fu-Sheng Wang

**Affiliations:** Department of Infectious Diseases, Fifth Medical Center of Chinese PLA General Hospital, National Clinical Research Center for Infectious Diseases, 100039 Beijing, China

**Keywords:** Infectious diseases, Stem-cell research

## Abstract

The coronavirus disease 2019 (COVID-19), caused by severe acute respiratory syndrome coronavirus 2 (SARS-CoV-2), has placed a global public burden on health authorities. Although the virological characteristics and pathogenesis of COVID-19 has been largely clarified, there is currently no specific therapeutic measure. In severe cases, acute SARS-CoV-2 infection leads to immune disorders and damage to both the adaptive and innate immune responses. Having roles in immune regulation and regeneration, mesenchymal stem cells (MSCs) serving as a therapeutic option may regulate the over-activated inflammatory response and promote recovery of lung damage. Since the outbreak of the COVID-19 pandemic, a series of MSC-therapy clinical trials has been conducted. The findings indicate that MSC treatment not only significantly reduces lung damage, but also improves patient recovery with safety and good immune tolerance. Herein, we summarize the recent progress in MSC therapy for COVID-19 and highlight the challenges in the field.

## Introduction

The coronavirus disease 2019 (COVID-19) caused by severe acute respiratory syndrome coronavirus 2 (SARS-CoV-2) has affected over 184 million patients and caused more than 3.98 million deaths up to July 6, 2021, with these numbers continuously increasing.^1^ SARS-CoV-2 causes a spectrum of clinical manifestations, ranging from mild or moderate respiratory symptoms to severe acute respiratory syndrome (SARS) and death. Patients with acute SARS-CoV-2 infection often present a constellation of symptoms similar to their counterparts with SARS, middle East respiratory syndrome, and influenza.^2,3^ The host’s innate and adaptive immune responses, especially specific adaptive immunity to SARS-CoV-2, play an essential role in controlling viral infection.^4^ Excessive inflammation and the cytokine storm are regarded as major causes of organ damage that drive the progression of severe COVID-19.^5–8^ Therefore, aside from direct antiviral treatment and supplemental oxygen therapy for COVID-19 cases,^4^ immunomodulatory therapeutic strategies may potentially prevent disease progression and rescue COVID-19 patients, especially in cases of severe and critical illness. Many immunotherapeutic approaches have been used for COVID-19, including glucocorticoid therapy, convalescent plasma therapy, and anti-interleukin (IL-6) receptor antibody therapy.^4,9–11^ However, the side effects and variable treatment efficacy have necessitated further studies to identify the safety and effectiveness of alternative immune-modulation regimens.

The safety and efficacy of mesenchymal stem cell (MSC) therapies have been recently illustrated in clinical trials, such as immune-mediated inflammatory diseases as systemic lupus erythematosus^12^ and graft-versus-host disease (GVHD).^13^ Notably, MSC treatment has been used in influenza-infected animal models and patients, significantly inhibiting the immune cell-mediated inflammatory response and reducing further lung damage.^14,15^ MSCs have also been selected to treat patients with acute respiratory distress syndrome (ARDS).^16–19^ Since the outbreak of COVID-19 pandemic in January 2020, a series of phase-1 and phase-2 clinical trials of mesenchymal stem-cell therapy projects have been launched by our team. To date, more than 60 stem-cell clinical trials for COVID-19 therapy have been registered at ClinicalTrials.gov. This perspective mainly focuses on these stem-cell–based approaches, summarizes the current progress, and discusses the challenges in this field.

## Rationale for stem-cell therapies

### Pathology and pathogenesis of COVID-19

For the first time, our team described the pathological characteristics of a patient who died of critical COVID-19. Bilateral diffuse alveolar injury was observed in the lungs, accompanied by cellular fibrous mucinous exudates and ARDS.^20^ Interstitial mononuclear inflammatory infiltration, dominated by lymphocytes, was widespread in both lungs. Multinucleated syncytial cells were observed in the alveoli, characterized by prominent nucleoli, amphiphilic granular cytoplasm, and large nuclei showing viral cytopathic-like changes. In other studies, distribution of SARS-CoV-2 in multiple extrapulmonary organs has been reported in COVID-19 patients.^21^ Kidney injury and myocardial infarction represent the pathological characteristics of critically illness.^22–24^

SARS-CoV-2 infection directly leads to immune disorder in both the adaptive and innate immune responses.^25,26^ Inflammatory and immune profiles in patients with COVID-19 have been well characterized.^7,27^ Specifically, the proportions of natural killer (NK) cells, CD4 + T cells, and CD8 + T cells significantly decrease.^26,27^ Moreover, T cells show an enhanced migration ability and acute inflammatory response, accompanied by a significantly increased expression of inhibitory molecules, while the naive T compartment is reduced.^27^ The percentage of plasma B cells and B-cell clonality are increased.^27–29^ The innate immunity components also show disturbance. The proportions of dendritic cell compartments are significantly decreased, while the IFN response profiles are elevated. In addition, γδ T cells, NK cells, and CD16 + monocytes are significantly activated.^27^

### Immunomodulation and therapeutic principles of MSCs

Stem cells are classified as either embryonic or adult, based on their source of isolation or origin. The use of embryonic stem cells is restricted because of religious, ethical, and legal controversies. Currently, MSCs are most widely utilized in clinical practice because of their ability to escape recognition by immunocytes, owing to their minimal expression of class-II molecules of the major histocompatibility complex.

**Fig. 1:**
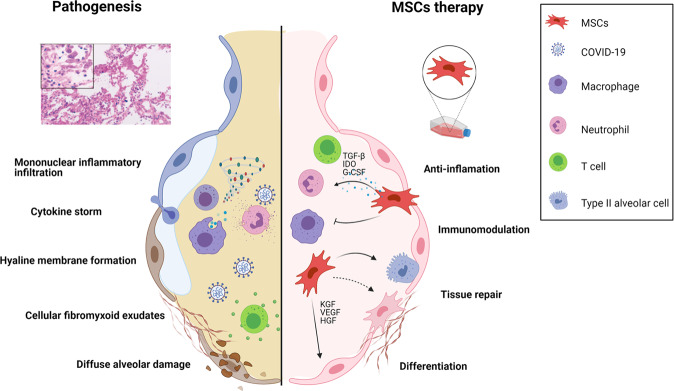
Proposed mechanisms for MSC action in patients with severe COVID-19. At first, SARS-CoV-2 primarily occupies the respiratory tract including the lung; the infiltration of immune cells (neutrophils, monocytes/macrophages, NK, CD4 + T, CD8 + T, Th17, and B cells) increases; then cytokine storms (including IFN-α, IL-1, IL-6 and TNF-α) occur. Hyaline membrane formation, the release of cellular fibromyxoid exudates, and pneumocyte desquamation are also observed. After stem-cell infusion, the number of infiltrated immune cells decreases significantly, and the damaged lung tissue is repaired. MSCs play a role in regeneration and immune regulation, but the detailed mechanisms underlying these effects remain to be fully elucidated

MSCs are non-hematopoietic cells which can be isolated from various sources, including umbilical cord, adipose tissue, bone marrow, and human dental pulp.^30,31^ MSCs have differentiational and regenerative properties and can secrete hepatocyte growth factor, vascular endothelial growth factor, and keratinocyte growth factor to promote the regeneration of type II alveolar epithelial cells.^32^ Furthermore, MSCs can be attracted to sites of inflammation by different chemokines and exert the potential to modulate the functions of various immunocytes—such as NK cells, dendritic cells, B cells, T cells, neutrophils, and macrophages—through direct contact and paracrine effects. Indoleamine 2,3-dioxygenase, transforming growth factor β, human leukocyte antigen isoform, and prostaglandin E2 have been identified as the major effectors.^33^ Thus, MSCs may offer a therapeutic option for patients with severe or critical COVID-19, potentially contributing to recovery from lung damage, suppressing the over-activated inflammatory response, and influencing the progression of pulmonary fibrosis. In both animal models and humans, MSC treatment has been observed to reduce pulmonary lesions and inhibit the inflammatory response induced by influenza virus infection.^14,15^ The potential efficacy and safety of MSC treatment have also been evaluated in patients with ARDS.^16–19^ Types of stem cell other than MSC,^34^ such as human embryonic stem cell-derived immunity- and matrix-regulatory cells (hESC-IMRCs),^35^ have also been employed to treat COVID-19 patients (Fig. 1). MSC preparation and quality control must comply with drug production implementation standards based on total quality management requirements. Before clinical treatment, stem cell preparations should be sent to authorized third-party institutions for strict quality inspection.

## Clinical trials of MSC therapies for COVID-19

### Phase-1 trials

Recent data from phase-1 clinical trials have demonstrated that the intravenous transfusion of MSCs in moderate or severe COVID-19 patients was safe and well-tolerated. Zhao et al.^36^ reported for the first time that intravenous administration of MSCs could improve the clinical outcome of COVID-19 patients while demonstrating good levels of immune tolerance, especially in critically ill patients. Li et al.^37^ reported a trial using menstrual blood-derived MSCs (MB-MSCs) for severe patients, which indicated that MSC transplantation might serve as an alternative option for treating COVID-19, particularly in critical patients. In our study,^38^ we found that umbilical cord MSC (UC-MSC) infusion in moderate or severe COVID-19 patients was safe, paving the way for phase-2 and -3 trials with 96 weeks of follow-up. In addition, a high dose of MSCs (200 × 10^6^ cells)^39^ and exosomes derived from allogeneic MSCs^40^ were evaluated for treatment efficacy in COVID-19 patients; this treatment was well-tolerated and showed potential improvement in some clinical parameters.^41–44^ Very recently, Mirakaj et al. found that patients who underwent MSC treatment, compared against control patients, experienced significantly lower lung damage (Murray score) upon discharge, had a higher survival rate to discharge, and had better recovery of pulmonary functions.^20,45,46^ Apart from MSCs, Wu et al.^35^ showed that lung fibrotic lesions were decreased by transfusion of hESC-IMRCs in patients with pulmonary fibrosis.

### Phase-2 trials

We performed a randomized, double-blind, placebo-controlled phase-2 trial to evaluate the efficacy and safety of intravenous MSC treatment in two hospitals in Wuhan, China.^47^ 101 patients with severe COVID-19 were recruited and assigned randomly at a 2:1 ratio to receive UC-MSCs or a placebo, respectively. Compared with the placebo, UC-MSC transfusion exerted a improvement in lung lesion volume from baseline to day 28. UC-MSCs also reduced the proportion of solid-component lesion volume significantly, and the 6 min walking distance (6MWD) showed an increased distance in the MSC group. The incidence of adverse events and severe adverse events was similar between the groups. Lanzoni et al. performed another double-blind, randomized, controlled trial and found that UC-MSC infusions significantly decreased cytokine levels at day 6 and improved survival in COVID-19 patients with ARDS.^48^ In this trial, 24 subjects were randomized and assigned 1:1 to receive either MSCs or placebo. MSC medication was associated with a significant improvement in the survival rate without serious adverse events. These data suggest that MSC treatment is safe and may be beneficial for COVID-19 patients (Table 1).

**Table 1:** Clinical studies of MSCs treatment for patients with COVID-19

Reference/Trial ID	Trial design	Indications	Source of MSCs	Dose of MSCs	Route and time of administration	Number of patients	Findings
ChiCTR2000029606 Tang L, et al.^37^	Case report	ARDS	Allogeneic, menstrual blood-derived	1 × 10^6^ cells/kg,	IV 3 rounds (day 0,1,3)	2	Fraction of inspired O_2_ (FiO2) and partial pressure of oxygen (PO2) improved, well-tolerated.
ChiCTR2000029990 Leng Z, et al.^36^	Phase 1, open label, single center, Case-control	Moderate/Severe/Critical	MSCs	1 × 10^6^ cells/kg,	IV 1 round	10	The pulmonary function and symptoms were significantly improved after MSC transplantation.
NCT04252118 Meng F, et al.^38^	Phase 1, open label, single center, case-control	Moderate/Severe	Umbilical cord	3 × 10^7^ cells/dose	IV 3 rounds (day 0, 3 and 6).	18	Intravenous UC-MSCs infusion in moderate and severe COVID-19 patients is safe and well-tolerated.
NCT04348461 Sánchez-Guijo F, et al.^44^	Phase 1, prospective nonrandomized open-label cohort	Severe/Critical	Adipose-derived	1 × 10^6^ cells/kg	IV 1, 2 or 3 rounds	13	No adverse events were reported. Improvement in ventilatory, radiological and biological parameters was associated with clinical response.
ChiCTR2000031494 Shu L, et al.^46^	Phase 1, open-label, randomized, standard treatment-controlled trial	Severe/Critical	Umbilical cord	2 × 10^6^ cells/kg	IV 1 round	41	MSCs induced the clinical improvement.
National Medical Products Administration of China Wu J, et al.^35^	Phase 1, prospective, nonrandomized, open-label cohort	COVID-19 patients with lung fibrosis	hESC-IMRCs	3 × 10^6^ cells/kg	IV 1–3 rounds	27	Lung fibrosis lesions were decreased.
IRCT20200217046526N2 Hashermian SR, et al.^39^	Phase 1	ARDS	Umbilical cord and placental	2 × 10^8^ cells/round	3 rounds ((day 0, 2 and 4).	11	MSCs can improve respiratory distress and reduce inflammatory biomarkers in some critically illness with well tolerance.
NCT04355728 Giacomo Lanzoni, et al.^48^	Double‐blind, randomized, phase 1/2a, trial	ARDS	Umbilical cord	10 ± 2 × 10^7^ cells/round	IV 2 rounds (day 0 and 3)	24	UC‐MSC infusions were safe. Inflammatory cytokines were decreased significantly, and patient survival was improved.
NCT04288102 Shi L, et al.^47^	Phase 2, randomized, double-blind, placebo-controlled trial.	Severe	Umbilical cord	4 × 10^7^ cells/dose	IV 3 rounds (day 0, 3 and 6).	100	UC-MSCs administrations were safe and exerted improvement in whole lung lesion volume compared with the placebo, especially in solid-component lesion. The 6MWD showed an increased distance in patients treated with UC-MSCs.
Local ethics approval Helene Helene Häberle, et al.^45^	Phase 1	Severe COVID-19 ARDS	Bone marrow-derived mononuclear cells	NA	NA	23	MSC infusion was safe. The MSC group had a significantly higher Horovitz score on discharge than the control group.

*MSC* mesenchymal stem cell, *ARDS* acute respiratory distress syndrome, *hESC-IMRCs* human embryonic stem cell–derived immunity-and matrix-regulatory cells, *IV* intravenous injection, *NA* not available

## Current challenges

A major challenge for mesenchymal stem cell therapy is the need to confirm its efficacy in controlling pulmonary fibrosis by means of a multi-cohort, randomized, controlled trial with a long-term follow-up. In addition, it is important to standardize MSC products and their clinical application protocols. In the United States, the US Food and Drug Administration (US FDA) classifies cells, tissues, or products based on cells and tissues (HCT/Ps) in the context of defining cell-therapy products as pharmaceutical methods. The US FDA is developing protocols to reliably evaluate and characterize MSC products for safety and effectiveness.^49^ In the European Union, advanced therapy products are classified and supervised, with the European Medicines Agency focusing on the biological characteristics and scope of clinical application for disease prevention, diagnosis, or treatment, and emphasizing treatment. In China, clinical trials involving stem cells must be performed according to *The Guidelines for Quality Control and Preclinical Study of Stem Cell Preparations (Trial)* and *The Stem Cell Clinical Research Management Methods* issued by the National Medical Products Administration and the National Health Protection Commission, to ensure that MSC therapy trials are performed in a scientifically rigorous manner according to international standards.^50^

Standardization of stem-cell clinical application protocols may directly affect treatment efficacy: the right regimen for the right patient at the right time. Key areas to optimize include the type of MSCs to be transfused, whether the cells are fresh or frozen (and their viability before infusion), the administration regimen (including dosage, interval, and number of cycles), the route of administration, and delivery of MSCs within a specific phase of the COVID-19 disease. Published results provide preliminary evidence for substantial efficacy and a high tolerance level, substantiating the need for larger, adjusted, and stratified phase-3 clinical trials in coming years. In addition, it is important to document the physiological alterations caused by MSCs in animal models to track the fate of the infused MSCs and elucidate the mechanisms that underlie the interactions between infused MSCs and the inflammatory microenvironment. If the above challenges can be resolved, the clinical application of MSCs for COVID-19 patients—with controllable and feasible standards—will benefit patients and provide an additional emergency capacity.

## Perspective and strategies

MSC therapies provide a promising and challenging opportunity for patients with COVID-19. These therapies appear to be optimal candidates for ameliorating inflammation, contributing to lung-tissue recovery, preventing long-term pulmonary disability, and reducing mortality. Phase-3 trials are still necessary for further evaluation of the efficacy and associated mechanisms of MSC treatment.

China has adopted reasonable measures to combat the spread of SARS-CoV-2. However, in some countries the pandemic is not well controlled, even exhibiting a trend of increasing spread of the disease; this is particularly the case in countries with limited medical resources and insufficient access to vaccines. We believe that efficient vaccination strategies will ultimately control the COVID-19 pandemic. The development of stem-cell therapy will benefit patients with pulmonary damage resulting from other viral and nonviral diseases such as post-traumatic ARDS, lung GVHD, and diseases characterized by hyper-inflammatory and hyper-immune reactions.
